# Single Pd Atoms on θ-Al_2_O_3_ (010) Surface do not Catalyze NO Oxidation

**DOI:** 10.1038/s41598-017-00577-y

**Published:** 2017-04-03

**Authors:** Chaitanya K. Narula, Lawrence F. Allard, Melanie Moses-DeBusk, G. Malcom Stocks, Zili Wu

**Affiliations:** 1 Materials Science & Technology Division, Oak Ridge National Laboratory, Oak Ridge, TN 37831-6133 USA; 2 Energy & Transportation Science Division, Oak Ridge National Laboratory, Oak Ridge, TN 37831 USA; 3 Chemical Sciences Division, Oak Ridge National Laboratory, Oak Ridge, TN 37831 USA

## Abstract

New convenient wet-chemistry synthetic routes have made it possible to explore catalytic activities of a variety of single supported atoms, however, the single supported atoms on inert substrates (e.g. alumina) are limited to adatoms and cations of Pt, Pd, and Ru. Previously, we have found that single supported Pt atoms are remarkable NO oxidation catalysts. In contrast, we report that Pd single atoms are completely inactive for NO oxidation. The diffuse reflectance infra-red spectroscopy (DRIFTS) results show the absence of nitrate formation on catalyst. To explain these results, we explored modified Langmuir-Hinshelwood type pathways that have been proposed for oxidation reactions on single supported atom. In the first pathway, we find that there is energy barrier for the release of NO_2_ which prevent NO oxidation. In the second pathway, our results show that there is no driving force for the formation of O=N-O-O intermediate or nitrate on single supported Pd atoms. The decomposition of nitrate, if formed, is an endothermic event.

## Introduction

The introduction of automotive catalysts in 1971 ushered in a new era of successful commercialization of supported catalysts on a very large scale^1^. Since then, advances in emission treatment catalysis have enabled automobile manufactures to meet stringent regulatory requirements for CO, NO_x_, and hydrocarbon emissions. Typically, automotive catalysts contain platinum dispersed on alumina, and rhodium dispersed on ceria-zirconia. Palladium has been successfully employed to substitute for platinum. Generally, fresh catalysts contain single atoms, rafts, nanoparticles with no defined structure, and well-defined discrete crystalline particles that range from nanoparticles to large particles. The gradual but persistent sintering during use decreases the number of single atoms, rafts, and nanoparticles and increases the number of large particles^1, 2^.

Reduction of NO_x_ and oxidation of CO and hydrocarbons occurs simultaneously in catalysts that treat emissions from a stoichiometric engine. The catalytic reactions for CO, NO_x_, and hydrocarbon conversion occur via Langmuir-Hinshelwood (L-H) pathways which require adsorption of reactive gases on metal surfaces leading to redox reactions^1, 3^. The decrease in metal particle size generally increases catalyst performance^4–6^. Theoretical and experimental studies have shown that sub-nanometer particles are more effective than nanosized particles^7–13^. However, this is not the case for NO oxidation which decreases with decrease in particle size. The anomalous behavior of NO is due to oxidation of small metal particles which prevent facile NO adsorption^14–17^. Thus, a balance in particle size is necessary to achieve optimum performance from emission treatment catalysts.

The smallest metal “particles” are single supported metal atoms which are also present in fresh catalysts. Until recently, it was unknown whether they participate in catalysis or are mere spectators waiting to be sintered. Recent progress clearly shows that single supported atoms are catalytically active towards a variety of reactions^18–20^. The development of solution methods for the synthesis of supported single atoms facilitated the study of catalytic activity^21–30^. The work from Abbet *et al*. showed that single Pd atoms supported on an MgO surface are catalytically active for CO oxidation^31^. Since the conventional L-H pathway is not possible on single supported Pd atoms, a modified L-H pathway, involving the substrate was proposed by the authors. For single supported Pd on γ-alumina, two types of structure have been proposed. The surface Pd adatoms bound to four-fold hollow sites on a (100)-terminated alumina has been described by Lee *et al*.^22^ for mesoporous alumina supported Pd while cationic structure has been reported by Datye *et al*.^29^ for Pd impregnated on commercial γ-alumina.

We have recently shown that inert substrate supported single Pt-atoms^32, 33^ are catalytically active for both CO and NO oxidation. Our results also showed that single Pt atoms are as effective as Pt particles for NO oxidation and do not follow the trend of decrease in NO oxidation with decrease in Pt particle size. Intrigued by a recent report from Datye *et al*.^29^ that atomic Pd on γ-alumina can oxidize CO effectively at room temperature, we carried out NO oxidation on single Pd atoms supported on θ-alumina. The rationale for employing θ-alumina instead of γ-alumina in preparation of model catalyst has been presented previously^32, 33^. Although Pd particles are not as effective as Pt particles for NO oxidation^34–37^, we were surprised to find no detectable NO oxidation under our experimental conditions. We also carried out *in-situ* DRIFTS studies to gain insights into NO interaction with single supported Pd atoms. In our previous work on single supported Pt atoms, we found strong nitrate bands (in comparison to alumina substrate only) under NO oxidation conditions. We reasoned that the presence of nitrate peaks, stronger than substrate, could be indicative of NO oxidation. However, the nitrate peaks were weaker on single supported Pd atoms than those on alumina substrate.

The proposed mechanistic pathways for oxidation reactions on single supported atoms are essentially modified L-H pathways and either involve the substrate as an oxygen source or the reaction proceeds with both oxygen and reactive gases adsorbed on the metal atom. For example, CO oxidation in the first pathway occurs via CO adsorption on metal atom which then reacts with oxygen from substrate to eliminate CO_2_. In the second pathway, CO and oxygen are adsorbed on metal atom and form either O-O-C=O or carbonate intermediate which then eliminates CO_2_. Most of the known examples of single supported atoms (Pt, Ir, Au) have bene prepared on active substrates such as CeO_2_ or FeO_x_ and the proposed modified Langmuir-Hinshelwood [L-H] pathways that utilize substrates as oxygen sources have been shown to be energetically favorable^21–26^. The exceptions are the single atoms supported on alumina clusters (e.g. PtAl_3_O_7_^−^) which are highly reactive towards CO oxidation but follow a molecular pathway where oxygen is supplied by alumina cluster for CO_2_ formation and then alumina cluster re-oxidizes in oxygen^27, 28^. Alumina has also been proposed to provide oxygen for CO oxidation reactions over atomic Pd^29^.

Recent detailed theoretical study of NO oxidation on Pd and other metals explores the role of oxygen-coverage, cluster sizes, metal-oxygen binding strength, and oxygen dissociation^36^. For Pd, palladium oxide is believed to be active catalyst for NO oxidation^38–40^. However, such a study does not exist for single supported Pd atoms. As such, we explored both pathways, commonly suggested for oxidation on single supported atoms, for NO oxidation on single Pd atoms. First pathway is analogous to the one described by Datye *et al*.^29^ for CO oxidation, involves NO adsorption on Pd, and employs alumina as oxygen source. The results show that there is energy barrier for the release of NO_2_ suggesting that NO oxidation will not be facile. We also explored the second pathway where both NO and oxygen are adsorbed on Pd atom and reaction proceeds via O=N-O-O or NO_3_ intermediates. We find that there is energy barrier to the formation of O=N-O-O or NO_3_ intermediates and there is no driving force to release NO_2_.

## Results and Discussion

**Figure 1:**
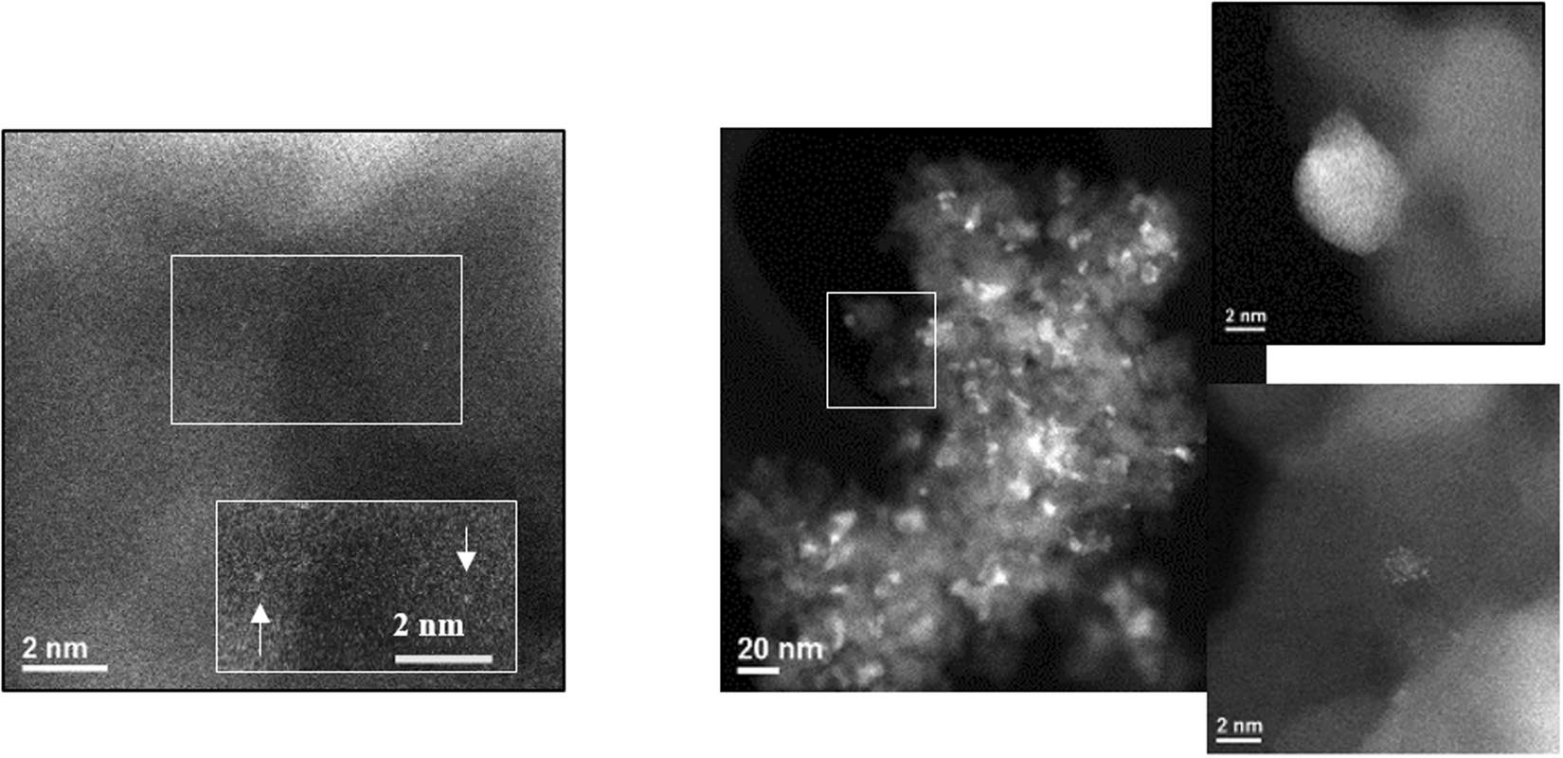
HAADF-STEM images of (I) Pd_s_-θ-alumina and (II) Pd-θ-alumina (right). For Pd_s_-θ-alumina, the inset is an enlargement of the boxed area after additional contrast and brightness was used to make the single atoms more apparent. For Pd-θ-alumina, the inset has been magnified to observe particles and rafts.

The θ-Al_2_O_3_ supported single Pd atom and Pd particles are named Pd_s_/θ-Al_2_O_3_ and Pd/θ-Al_2_O_3_, respectively. The synthesis of θ-alumina supported Pd samples, Pd_s_/θ-Al_2_O_3_ and Pd/θ-Al_2_O_3_, was carried out by impregnating θ-alumina powder with tetra-ammine palladium nitrate and subsequent sintering at 700 °C. The Pd loading was kept at 0.2% to obtain mono-disperse palladium single atoms (Pd_s_/θ-Al_2_O_3_) and at 2% (Pd/θ-Al_2_O_3_) to obtain rafts. HAADF-STEM images of fresh Pd_s_/θ-Al_2_O_3_ show single Pd atoms only [Fig. 1, left] and that of Pd/θ-Al_2_O_3_ exhibit particles and rafts [Fig. 1, right].

### NO Oxidation

**Figure 2:**
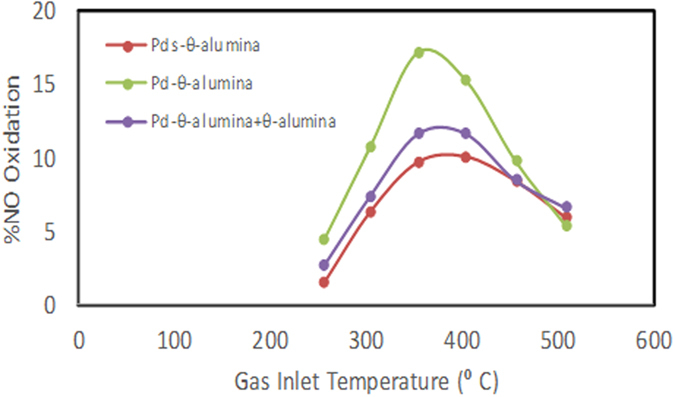
The NO oxidation light-off over Pd_s_-θ-alumina and Pd-θ-alumina employing 420 ppm NO + 5% O_2_. NO conversion over pure θ-Al_2_O_3_ is not shown since it overlaps with the plot for Pds/θ-Al_2_O_3_.

The NO oxidation for both Pd_s_/θ-Al_2_O_3_ and Pd/θ-Al_2_O_3_ were carried out under two conditions, 420 ppm NO + 5% O_2_ [Fig. 2] and 50 ppm NO + 1% O_2_ [Figure S1]. For comparison, we also carried out NO oxidation on pure θ-alumina. The NO oxidation over Pd_s_/θ-Al_2_O_3_ is about same as that over θ-alumina in the 250 to 510 °C inlet gas temperature range. The Pd/θ-Al_2_O_3_ catalyst exhibits 17% NO conversion at 354 °C inlet gas temperature, which is about twice that of θ-Al_2_O_3_ [Fig. 2]. The NO conversion over Pd/θ-Al_2_O_3_ mixed with θ-alumina to reduce Pd loading to 0.2% is 11% at 354 °C gas inlet temperature which is higher than both pure alumina and Pd_s_/θ-Al_2_O_3_.

When feed gas contains 50 ppm NO, the NO oxidation decreases to ~11% over Pd/θ-Al_2_O_3_ at 350 °C. Both θ-Al_2_O_3_ and Pd_s_/θ-Al_2_O_3_ show almost identical NO oxidation in 250 to 510 °C inlet gas temperature range [Figure S1]. A comparison of NO oxidation activity over single θ-alumina supported Pt (0.18% Pt/θ-Al_2_O_3_ shown as Pt_s_/θ-Al_2_O_3_) and Pd (Pd_s_/θ-Al_2_O_3_) is shown in Figure S2. The NO oxidation activity over Pd_s_/θ-Al_2_O_3_ is identical to that over θ-alumina and significantly less than that over Pt_s_/θ-Al_2_O_3_.

**Figure 3:**
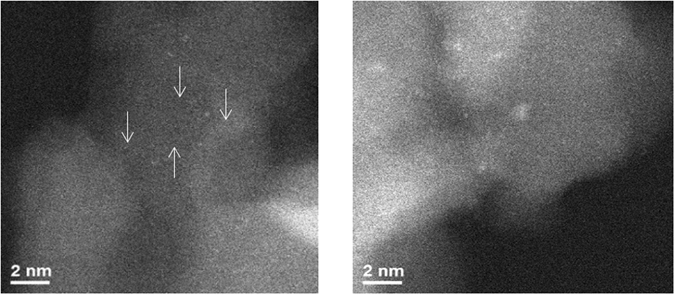
HAADF-STEM images of Pd_s_-θ-alumina post NO oxidation experiment show (**A**) single atoms and (**B**) small clusters.

The Pd_s_/θ-Al_2_O_3_ samples, after exposure to NO oxidation conditions, exhibit predominantly single atoms and ~1 nm clusters [Fig. 3]. No large particles were observed in any of the areas at low magnification. Since we did not observe any detectable NO oxidation on Pd_s_/θ-Al_2_O_3_, the very small particles are probably a result of thermal sintering. Since Pd/θ-Al_2_O_3_ is active for NO oxidation and NO is unable to induce Pd single atom sintering, the lack of observed NO oxidation on Pd_s_/θ-Al_2_O_3_ is due to the inability of Pd single atoms to oxidize NO. The Pd/θ-Al_2_O_3_ samples, after exposure to NO oxidation conditions does not undergo dramatic changes [Figure S3].

These experiments clearly show that Pd_s_/θ-Al_2_O_3_ is an ineffective NO oxidation catalyst and NO oxidation is not detectable over single supported Pd.

### *In-Situ* Diffuse Reflectance Studies

**Figure 4:**
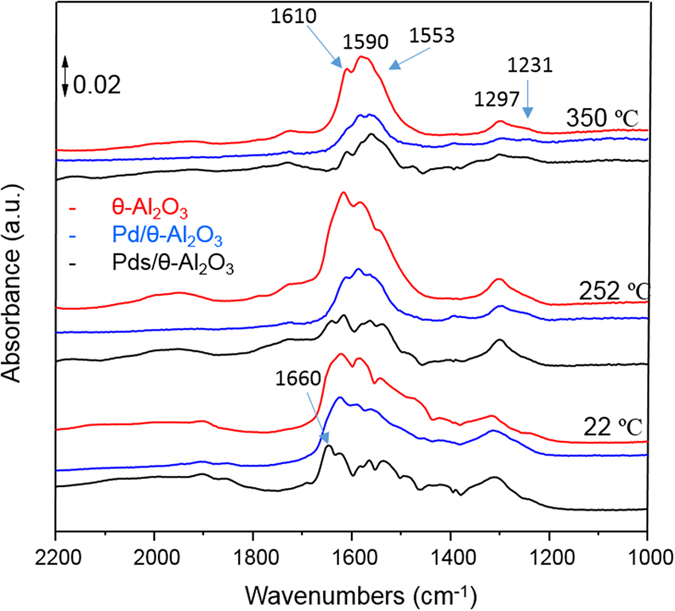
A comparison of *in situ* IR spectra during NO oxidation at 25, 250, and 350 °C on θ-alumina, Pds/θ-alumina, and Pd/θ-alumina.

We carried out *in-situ* DRIFTS studies to gain insights into NO interaction with single supported Pd atoms. The catalyst sample Pds/θ-Al_2_O_3_ was cleaned under oxidizing conditions employing a mixture of 5% oxygen in helium. The IR results for NO oxidation over θ-alumina and Pds/θ-alumina are shown in Fig. 4. At room temperature (~22 °C), the chelating bridging nitrate (N=O stretch at 1610 cm^−1^ and asymmetric NO_2_ stretch at 1553 cm^−1^) can be seen on θ-alumina^40^. Chelating bidentate nitrate is seen at 1590 cm^−1^ (N=O stretch) and 1297 cm^−1^ (NO_2_ asymmetric stretch) which grows stronger with increasing temperature and become strongest peaks at 350 °C^40^. Monodentate nitrate peaks (asymmetric NO_2_ at 1550 cm^−1^ and symmetric NO_2_ at 1279 cm^−1^), on the other hand, become weaker on increasing temperature and cannot be distinguished from chelating nitrate peaks at 350 °C^39^.

The IR spectra of Pds/θ-alumina during NO oxidation exhibit all the peaks that are seen for θ-alumina although intensity of all nitrate peaks is weaker than that on pure θ-alumina. Since the expected position of nitrate bonded to Pd in monodentate or bidentate mode (1616, 1553, 1312, 1279, 1231 cm^−1^) is close to those on θ-alumina, an increase in the intensity of nitrate bands would be an indication of formation of nitrates on palladium^41^. The DRIFTS of Pds/θ-alumina does give indication of Pd-NO^−^ at 1660 cm^−1^ at 22 °C which decreases in intensity at 252 °C and almost disappears at 350 °C^41^. These observations are very different from those previously reported for NO oxidation on single supported Pt atoms^33^. For Pts/θ-alumina the nitrate bands are stronger than those on pure θ-alumina at 252 °C in the absence of O_2_. Furthermore, the nitrates remain strong bands in the presence of NO + O_2_ at 300 °C. Thus, the nitrate formation is an essential step during NO oxidation over Pts/θ-alumina but is not observed for Pds/θ-alumina.

The IR spectra of Pd/θ-alumina during NO oxidation exhibits all the peaks that are seen for θ-alumina or Pds/θ-alumina at 22, 252, and 350 °C. Unlike Pds/θ-alumina, there is no indication of NO peaks at 1660 cm^−1^. The bridging nitrate peaks at 1616 and 1553 cm^−1^ are present at 22 °C but decrease with increase in temperature and essentially are a weak shoulder at 350 °C. The chelating nitrate peaks at 1590 and 1297 cm^−1^ increase with increase in temperature to 252 °C but decrease on further increase to 350 °C. Monodentate nitrate peaks at 1553 cm^−1^ increase in intensity with increase in temperature and can be distinguished from chelating nitrates.

Thus, the differences between inactive single Pd atoms and active Pd particles can be seen in the IR under NO oxidation conditions. First, NO peaks, present in Pd atoms are absent in Pd particles. Second, nitrate peaks are weak for Pd atoms probably due to effective nitrate decomposition but ineffective NO oxidation. Bridging nitrate peaks on active NO particles, on the other hand, decrease while monodentante and chelating nitrate increase with increase in temperature. Interestingly, the increase in nitrate peaks is not comparable to that observed for single Pt atoms^33^ which are far superior NO oxidation catalysts suggesting nitrate formation is an important step in NO oxidation.

### NO Oxidation on Pd/θ-alumina – *Theoretical Study*

#### Pd atom on Alumina Surface

**Figure 5:**
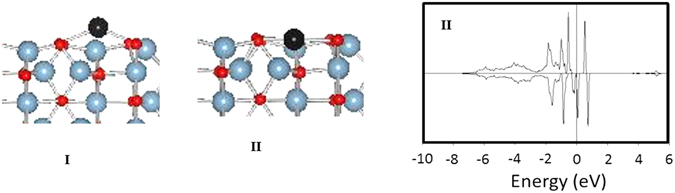
(I) Pd adatom^42^ and (II) Pd cation on θ-alumina (010) surface and its PDOS of d-orbitals.

A single Pd atom supported on a metal oxide surface can be present as either an adatom bonded to surface oxygen atoms [Fig. 5, I] or a cation replacing one of the surface aluminum atoms [Fig. 5, II]. We have previously reported the optimized structure of a Pd adatom on θ-alumina (010) surface^43^. Briefly, the Pd adatom occupies a positon above aluminum bonded to two surface oxygen atoms from two adjacent rows of the θ-alumina (010) surface^43^. The Pd atom is a d^10^ species with no magnetic moment associated with it. The Pd cation [Fig. 10, II top] resides in the alumina matrix and is bonded to 4 adjacent oxygen atoms with bond distances of 1.9, 2.06, 2.22, and 2.1 Å. The magnetization is associated with Pd and adjacent oxygen atoms bonded to Pd. The projected density of states [PDOS] analysis shows a vacancy in the dx^2^-y^2^ orbital suggesting a d^9^ species [Fig. 5, II bottom].

The optimized cationic Pd structure is similar to the one described for Pd cation on γ-alumina (100) surface^29^ with Pd exhibiting d^9^ oxidation state. Considering that a d^9^ Pd is rare and unstable, it is unlikely that it is representative of the single Pd atoms. Furthermore, it is unlikely that either of the two configurations represent single Pd atoms on alumina under ambient atmospheric conditions. In either case, the Pd will react with oxygen to form oxidized species.

**Figure 6:**
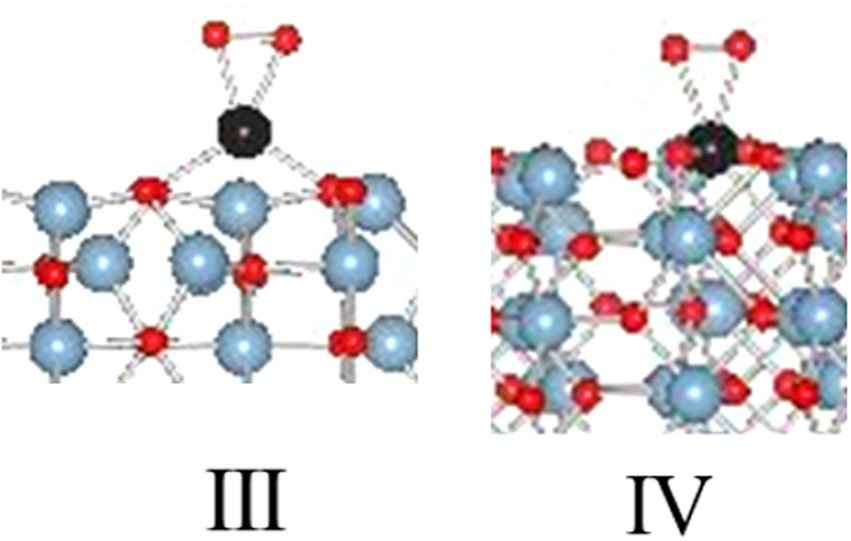
The interaction of single supported Pd atoms with oxygen.

The oxygen bonds to Pd adatom in a side-on (η2) mode to give configuration III via an exothermic step (−1.51 eV) [Fig. 6]. Our efforts to optimize structure with monodentate oxygen were unsuccessful as the oxygen gradually moved to bidentate mode of configuration III. For configuration III, the O-O bond distance is 1.39 Å and the projected densities of states (PDOS) show unoccupied 5d_xy_ [Figure S5] suggesting a d^8^ species. In addition, there is no magnetization associated with configuration III. This bonding mode is identical to the oxygen-bonding mode of Pd in organometallic complexes. For example, the X-ray structures of organometallic compounds, (R_3_P)_2_Pd(O_2_) have been determined previously and show oxygen in a η2 mode^42, 44–47^. These complexes exhibit an elongated O-O bond (1.41–1.48 Å) relative to dioxygen (1.21 Å) and superoxide (O^2−^) (1.33 Å) and approach the value for hydrogen peroxide (1.49 Å).

The Pd cation in configuration II interacts with oxygen in side-on η2-mode also resulting in configuration IV with Pd-O bond distances of 2.06 and 2.04 Å [Fig. 6]. The O-O bond distance is 1.308 Å and the O-Pd-O angle is 37.15°. The Pd now is bonded to six oxygen atoms and the magnetization is centered on the adsorbed oxygen atoms (μ_totalO2_ 0.64). The analysis of PDOS shows partially occupied dyz and dx2-y2 orbitals suggesting a Pd(II) species. Since the EXAFS data fit reported by Datye *et al*.^29^ show Pd bonded to four oxygen atoms (coordination number 4) under oxidizing conditions, configuration III might represent atomic Pd under ambient conditions more accurately.

### Pathway I. NO oxidation with alumina as an oxygen source

**Figure 7:**
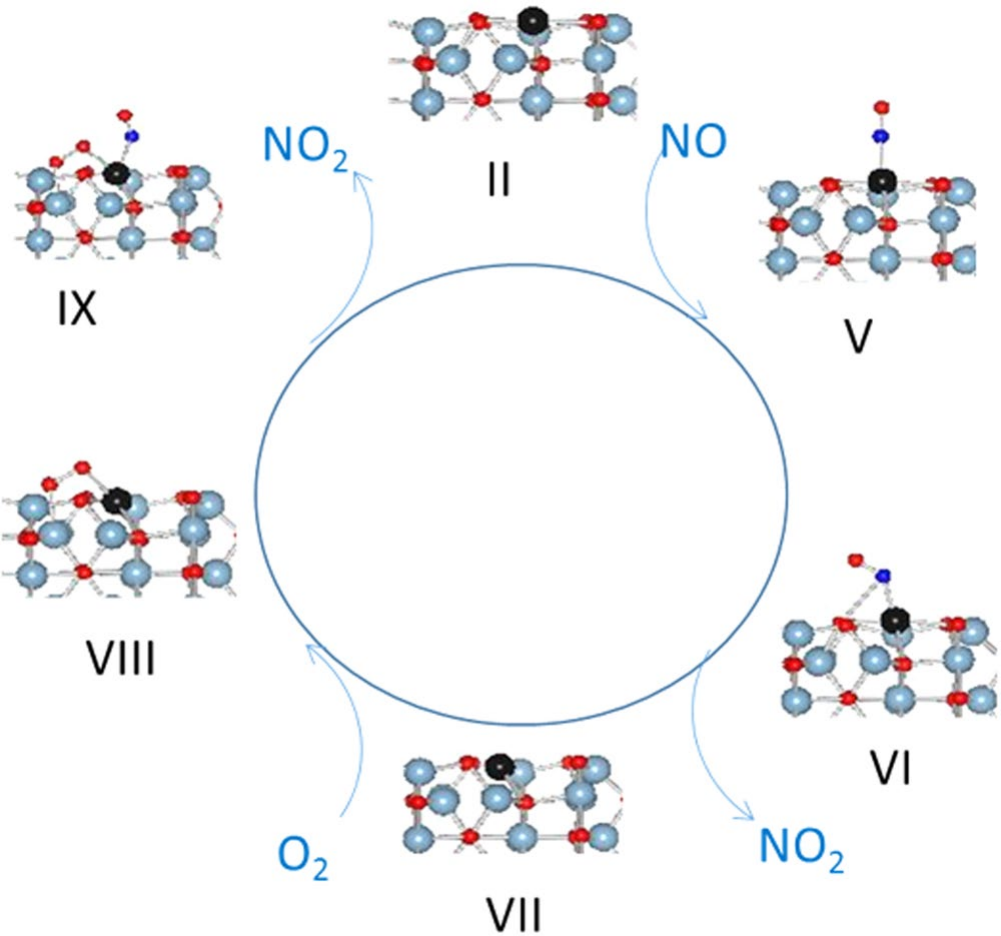
The NO oxidation cycle on a single supported Pd cation with alumina as the oxygen source.

This pathway [Fig. 7] is analogous to the mechanism recently reported for CO oxidation on single Pd atoms supported on γ-alumina and involves alumina as the oxygen source^29^. The total energy, Pd bond distances to surface oxygen, and magnetizations of intermediate configurations are summarized in Table S1.

This pathway assumes that Pd does not have molecular oxygen associated with it, and the NO oxidation initiates with NO adsorption on the Pd atom in configuration V. The NO adsorption is exothermic (−1.57 eV) and does not significantly impact the Pd bonds to surface oxygens (Table S1). The Pd-N and N-O bond distances are 1.83 and 1.16 Å respectively with a Pd-N-O angle of 172.9°. In this linear bonding mode, NO can be viewed as NO^+^ which is isoelectronic with CO. The magnetization is primarily associated with nitrogen and oxygen of the NO species. The PDOS analysis of Pd-d-orbitals shows partially filled dxy, dyx, and dx2 orbitals suggesting a d^8^ oxidation state [Figure S4, V].

The migration of NO in configuration V to form configuration VI results in the bending of NO bond resulting in a Pd-N-O angle of 136.5°. In this bonding mode NO is now NO^−^ and Pd also carries a slight magnetization. Loss of NO_2_ from configuration VI to configuration VII is an endothermic process. Configuration VII is essentially configuration II with one oxygen vacancy. In this configuration, Pd is bonded to two adjacent oxygen atoms, and the magnetic moment is carried over both oxygen atoms and the Pd atom. PDOS analysis shows partially occupied dyz and consequently a d^9^ Pd species [Figure S4]. Addition of molecular O_2_ to configuration VII leads to configuration VIII via an exothermic process. Magnetization in configuration VIII is primarily centered on Pd and an oxygen molecule. The PDOS shows partially occupied d-orbitals with components from all except d_x2-y2_ [Figure S4].

Addition of NO to configuration VIII via an exothermic process results in configuration IX. The NO bond is in a bent mode (Pd-N-O angle 152.6°) and is formally NO^−^. The magnetization is distributed over Pd, N-O, and all oxygen atoms bonded to Pd. The analysis of PDOS shows partially occupied dyz and dx2-y2 orbitals [Figure S4]. Finally, the release of NO_2_ from configuration IX via an exothermic process regenerates configuration II. The energetics of reactions in Fig. 7 are summarized as follows:

This mechanistic pathway suggests that there is a energy barrier to the release of first NO_2_ (−2.18 eV) although rest of the steps are energetically favorable. This contrasts with CO oxidation pathway, described by Datye *et al*.^29^ where CO_2_ release is an exothermic process.

$$\begin{array}{lllll}{}^{\ast }{\rm{P}}{\rm{d}}\,(\mathrm{II}) & +{\rm{NO}} & = & {}^{\ast }{\rm{P}}{\rm{dNO}}\,(V) & -1.57{\rm{eV}}\\ {}^{\ast }{\rm{P}}{\rm{dNO}}\,(V) &  & = & {}^{\ast }{\rm{P}}{\rm{dNO}}\,(\mathrm{VI}) & -0.13{\rm{eV}}\\ {}^{\ast }{\rm{P}}{\rm{dNO}}\,(\mathrm{VI}) & -{{\rm{NO}}}_{2} & = & {}^{\ast }{\rm{P}}{\rm{d}} \mbox{-} {\rm{O}}\,(\mathrm{VII}) & 2.18{\rm{eV}}\\ {}^{\ast }{\rm{P}}{\rm{d}} \mbox{-} {\rm{O}}\,(\mathrm{VII}) & +{{\rm{O}}}_{2} & = & {}^{\ast }{\rm{P}}{\rm{d}} \mbox{-} {\rm{O}}({{\rm{O}}}_{2})\,(\mathrm{VIII}) & -1.92{\rm{eV}}\\ {}^{\ast }{\rm{P}}{\rm{d}} \mbox{-} {\rm{O}}\,({{\rm{O}}}_{2})\,(\mathrm{VIII}) & +{\rm{NO}} & = & {}^{\ast }{\rm{P}}{\rm{d}} \mbox{-} {\rm{O}}({{\rm{O}}}_{2})(\mathrm{NO})\,(\mathrm{IX}) & -0.93{\rm{eV}}\\ {}^{\ast }{\rm{P}}{\rm{d}} \mbox{-} {\rm{O}}({{\rm{O}}}_{2})(\mathrm{NO})\,(\mathrm{IX}) & -{{\rm{NO}}}_{2} & = & {}^{\ast }{\rm{P}}{\rm{d}}\,(\mathrm{II}) & -0.98{\rm{eV}}\end{array}$$

### Pathway II. NO oxidation via NO and O_2_ adsorption on Pd

**Figure 8:**
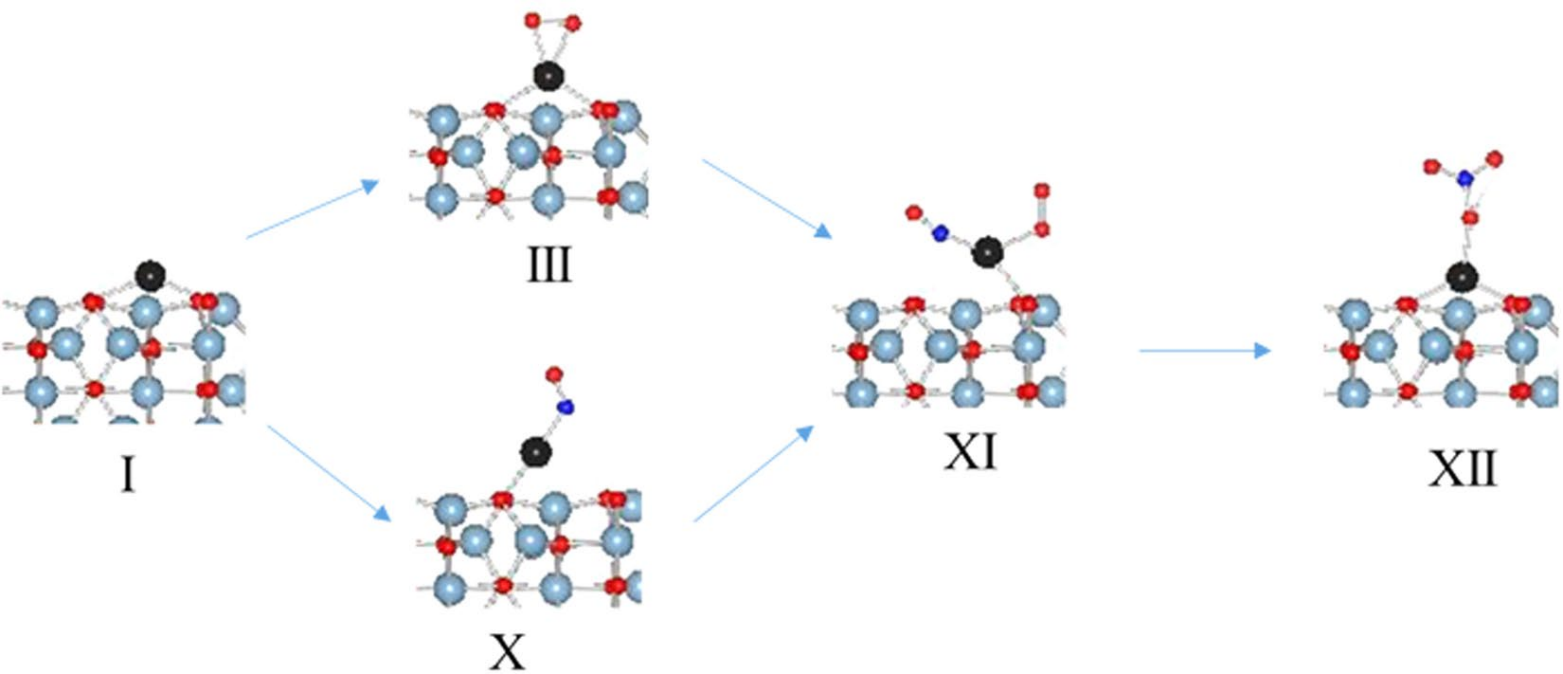
NO interaction with single supported Pd atom.

Since there is no significant difference between adsorption energies of O_2_ (−1.51 eV) or NO (−1.44 eV) on the Pd atom [Table S2], the NO oxidation can proceed with either the reaction of oxygen or NO on the Pd atom [Fig. 8]. However, a fresh catalyst can be expected to be oxidized (cf. configuration III) and the NO oxidation will proceed via NO adsorption on oxidized Pd in the first cycle. In subsequent cycles, the configuration I will form which can either react with NO or oxygen.

The NO bonds with the Pd adatom in configuration I via an exothermic reaction (−1.44 eV), and results in configuration X. The Pd-N-O angle is 125.9° suggesting NO is formally NO^−^ and is bonded to Pd in a bent mode. The Pd-N and N-O bond distances are 1.86 and 1.20 Å, respectively. One of the two surface Pd-O bonds breaks to accommodate the NO bonds to Pd. There is no magnetization associated with configuration IV, and PDOS analysis [Figure S5] shows filled d-orbitals. This suggests that Pd retains its zero oxidation state (d^10^).

Configuration XI can form via exothermic adsorption of NO in configuration III (−1.496 eV) or O_2_ on configuration X (−1.56 eV). In this configuration, molecular oxygen changes from η_2_ mode to terminal mode, and NO bonds to Pd in straight mode. Although there is a magnetic moment associated with configuration XI, all of the magnetic moment is localized on O_2_ (μO_2Total_, 0.86). One of the two Pd-O (surface) bonds breaks while the other slightly lengthens (0.2 Å). The Pd-N-O angle is 164.8° suggesting NO^+^ species, and the Pd-N bond is 1.867 Å.

**Figure 9:**
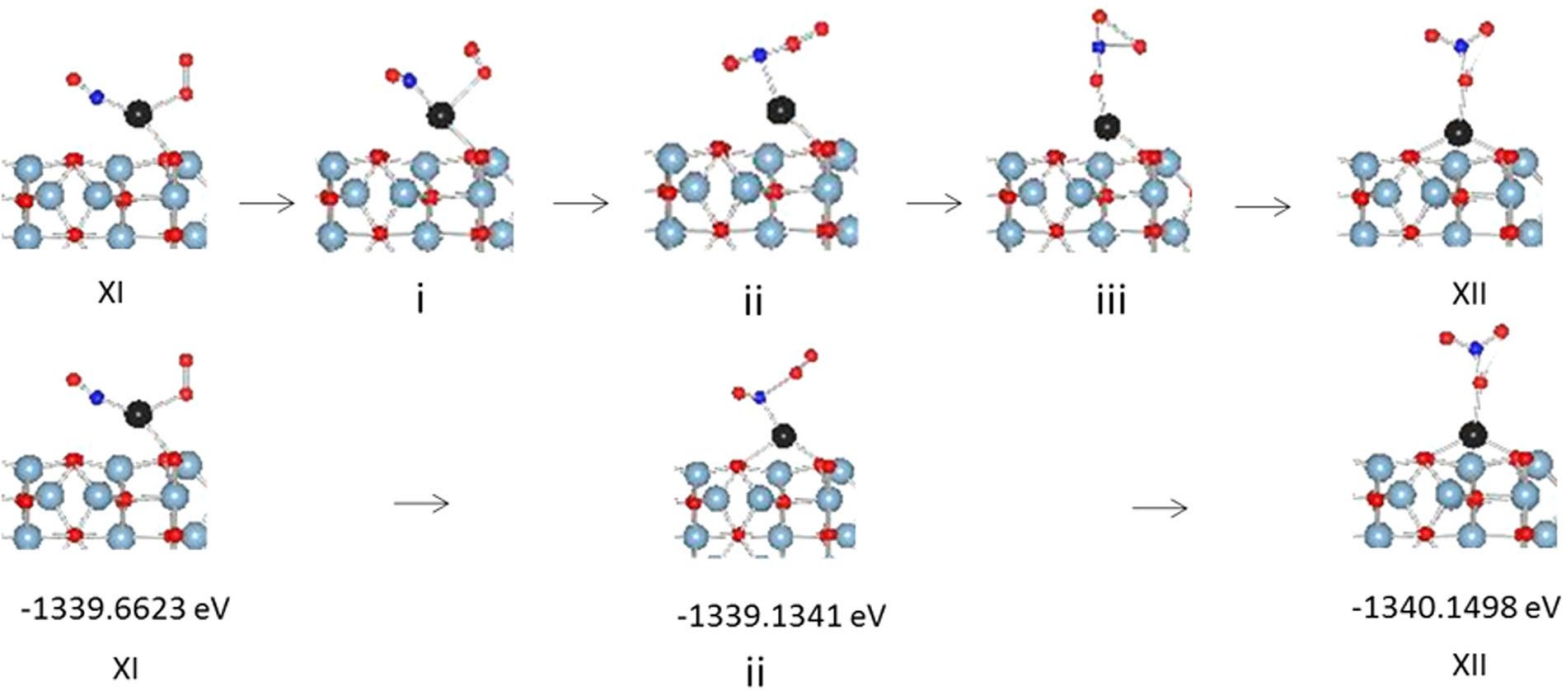
Transition States in nitrate formation.

**Figure 10:**
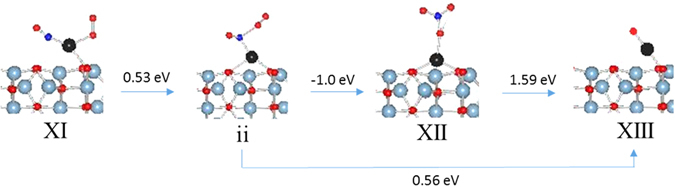
Energetics of NO_2_ release.

The transformation of configuration XI to configuration XII where nitrosyl forms a nitrate group is an exothermic process (−0.49 eV). The nitrate bonds in monodentate mode with the Pd atom and the Pd-O bond distance is 2.055 Å. The N-O bond where oxygen is bonded to Pd is slightly longer (1.324 Å) than the free N-O bonds of nitrate. The Pd-O surface oxygen bond lengths increase by 0.02 Å as compared with Pd-O surface oxygen bonds when Pd has no substituents. There is a magnetic moment associated with Pd and O from nitrate suggesting a d^9^ oxidation state for Pd. The PDOS analysis also shows unoccupied 5d_xy_ supporting a d^9^ oxidation state for Pd [Figure S5]. This structure is similar to nitrate complexes of Pd where nitrate bonds to Pd in monodentate mode^48, 49^. The three calculated transition states (i–iii) as well as optimized image ii are shown in Fig. 9. The image i optimized to a configuration which is very close to configuration XI in terms of structure and total energy while image iii optimized to a configuration close to configuration XII. The transformation of XI to transition state ii is a slightly endothermic step (0.53 eV) and directly release NO_2_ via another endothermic step (0.56 eV) to form configuration XIII [Fig. 10]. The transition state ii can form nitrate, XII, via an exothermic step (−1.0 eV). However, there is no driving force to form nitrate since XII is a d^9^ species which are rare and unstable. Furthermore, the release of NO_2_ from XII to from XIII is also an endothermic step (1.59 eV). Thus, there is no driving force to either form transition ii or the nitrate species XII and, if formed, the release of NO_2_ is an endothermic event.

If the reaction does proceed to configuration XII, the release of NO_2_ will lead to configuration XIII which can react with another molecule of NO to release second molecule of NO_2_ to complete the catalytic cycle. The details beyond configuration XII are presented in the Supplementary materials. Thus, this pathway shows that there is no driving force for the formation of O=N-O-O intermediate or nitrate on single supported Pd atoms. Furthermore, the decomposition of nitrate, if formed, is an endothermic event.

In conclusion, we have shown that single θ-alumina supported Pd atoms are completely ineffective for NO oxidation in contrast to single supported Pt atoms. The DRIFTS experiments show that nitrate peaks are even weaker than those on alumina under NO oxidation conditions suggesting that nitrate formation plays an important role in NO oxidation. To explain these results, we explored modified Langmuir-Hinshelwood type pathways that have been proposed for oxidation reactions on single supported atom. In the first pathway, we find that there is energy barrier for the release of NO_2_ which prevent NO oxidation. In the second pathway, our results show that there is no driving force for the formation of O=N-O-O intermediate or nitrate on single supported Pd atoms. The decomposition of nitrate, if formed, is an endothermic event.

## Methods

### Synthesis of Pd_s_/θ-Al_2_O_3_ and Pd/θ-Al_2_O_3_

The synthesis of Pd_s_/θ-Al_2_O_3_ and Pd/θ-Al_2_O_3_ was carried out by an impregnation method as described recently in literature^29^. The palladium loading for Pd_s_/θ-Al_2_O_3_ is 0.2% and that for Pd/θ-Al_2_O_3_ is 2%.

### Characterization of Catalysts

The samples were examined by transmission electron microscopy (TEM) and a Joel 2200FS FEG 200kv scanning transmission electron microscope (STEM) equipped with a CEOS GmbH (Heidelberg, Ger.) hexapole corrector on the probe-forming lenses. High-angle annular dark-field (HA-ADF) images showing single Pd atoms at a nominal resolution of 0.07 nm were collected at a 26.5 mr incidence semi-angle, using a detector having a 110 mr inner semi-angle.

### NO oxidation

NO oxidation experiments were carried out in two reactors. The GHSV was kept constant at 51.4 k h^−1^ for all tests. The feed gas (total flow 3.0 litres/min) composition was 50 ppm NO, 1% O_2_, and balance N_2_. The second set of experiments were carried out on a previously reported reactor that we employed to study NO oxidation on single supported Pt atoms^33^ to directly compare NO oxidation activity of single supported Pt and Pd. Briefly, the powder catalyst samples were loaded in a U-tube quartz reactor between two quartz wool plugs. To keep a constant gas hourly space velocity (GHSV) of ~55.5 k h^−1^, the palladium catalysts were mixed with blank θ-Al_2_O_3_ to provide 120 mg test samples (0.108 mL) for all tests. The feed gas composition was 50 ppm NO, 1% O_2_ and Ar as a balance. The catalysts were heated to 120 °C under Ar before the NO + O_2_ reaction gas was introduced and the reactor was further heated to the evaluation temperatures. Each sample was studied sequentially at the following catalyst bed set points: 270, 320, 420, and 520 °C. The catalyst was held at the set point and allowed to stabilize for ~70 min before the NO_X_ concentration was averaged over 5 min. The analyzer was switched to NO mode and given 1–2 minutes to stabilize, and then the NO concentration was averaged over the next 5 min. The NO oxidation activity was calculated as (NO_x_ − NO)/NO_x_.

### Infrared Study NO Adsorption

The detailed methods for NO oxidation by *in situ* diffuse reflectance Fourier transform infrared spectroscopy (DRIFTS) have been described previously^34, 35^. In summary, a Nicolet Nexus 670 spectrometer fitted with a MCT detector cooled with liquid nitrogen was used for recording spectra. The system is equipped with an *in-situ* chamber (HC-900, Pike Technologies) with a capability to heat samples to 900 °C. The NO oxidation samples were cleaned at 350 °C in a 5% O_2_ in helium. Samples were then cooled to experiment temperature under a flow of 5% O_2_ in helium. The NO oxidation experiments were carried out at 25, 250, and 350 °C by flowing a NO (2% NO in Ar, 5 mL/min) and O_2_ (5% O_2_ in He, 20 mL/min) mixture over the cleaned sample for 5 minutes followed by 5 minutes of O_2_ purging.

### Computational Method

The total energy calculations, based on ab initio DFT, were carried out employing the Vienna Ab Initio Simulation Package (VASP)^50^. A generalized gradient approximation (GGA) in the Perdew-Wang-91 form was employed for the electron exchange and correlation potential^51, 52^. The projector-augmented wave (PAW) approach for describing electronic core states was used to solve Kohn-Sham equations^52, 53^. The plane wave basis set was truncated at a kinetic energy cutoff of 500 eV. A Gaussian smearing function with a width of 0.05 eV was applied near Fermi levels. Ionic relaxations were considered converged when the forces on the ions were <0.03 eV/Å. We have previously described the details of construction of (010) alumina surface^49^. From bulk optimized θ-alumina, a 180-atom charge neutral 2 × 4 supercell was constructed^49^. The slabs were separated by a 15 Å vacuum to minimize spurious interaction by periodic images. A 4 × 1 × 4 Monkhorst-Pack mesh was used for surface calculations. Nudged elastic band method was employed to find transition states^54–56^.

## Electronic supplementary material

Supplementary Materials
