# Supplementary material for: Single Pd Atoms on θ-Al2O3 (010) Surface do not Catalyze NO Oxidation
Source: Sci Rep. 2017 Apr 3;7:560. doi: 10.1038/s41598-017-00577-y (PMC5428677; doi:10.1038/s41598-017-00577-y)
Supplement: Supplementary file 1 — Supplementary Materials [file 41598_2017_577_MOESM1_ESM.pdf]

Supplementary Materials

Single Pd Atoms on θ-Al_2O_3 (010) Surface do not Catalyze NO Oxidation

Chaitanya K. Narula,[1]* Lawrence F. Allard,[1] Melanie Moses-DeBusk,[2] G. Malcom Stocks, [1] and Zili Wu [3]

[1]Materials Science & Technology Division, Oak Ridge National Laboratory, Oak Ridge, TN, USA, 37831-6133

[2]Energy & Transportation Science Division, Oak Ridge National Laboratory, Oak Ridge, TN 37831

[3]Chemical Sciences Division, Oak Ridge National Laboratory, Oak Ridge, TN 37831

*Correspondence to: narulack@ornl.gov

This manuscript has been authored by UT-Battelle, LLC under Contract No. DE-AC05-00OR22725 with the U.S. Department of Energy. The United States Government retains and the publisher, by accepting the article for publication, acknowledges that the United States Government retains a non-exclusive, paid-up, irrevocable, world-wide license to publish or reproduce the published form of this manuscript, or allow others to do so, for United States Government purposes. The Department of Energy will provide public access to these results of federally sponsored research in accordance with the DOE Public Access Plan (http://energy.gov/downloads/doe-public-access-plan).

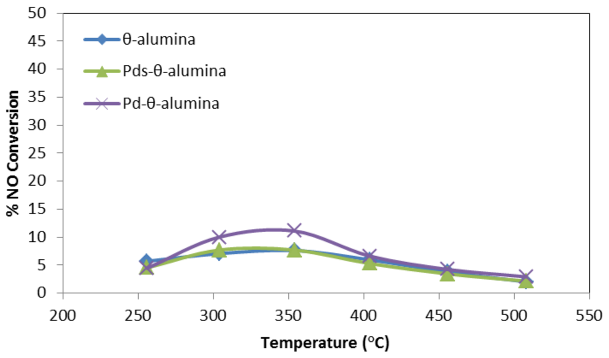

Figure S1. The NO oxidation light-off employing 50 ppm NO+1% O_2

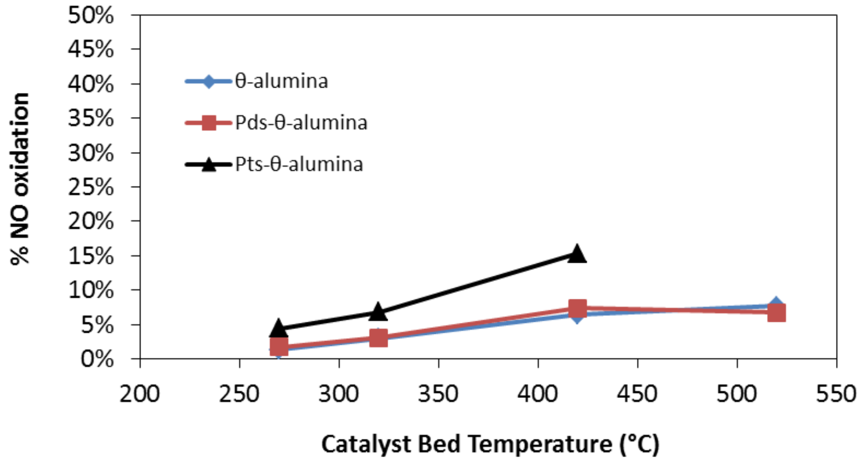

Figure S2. A comparison of NO oxidation over single θ-alumina supported Pt (Pt_s/θ-Al_2O_3) (from reference 26) and Pd (Pd_s-θ-alumina)

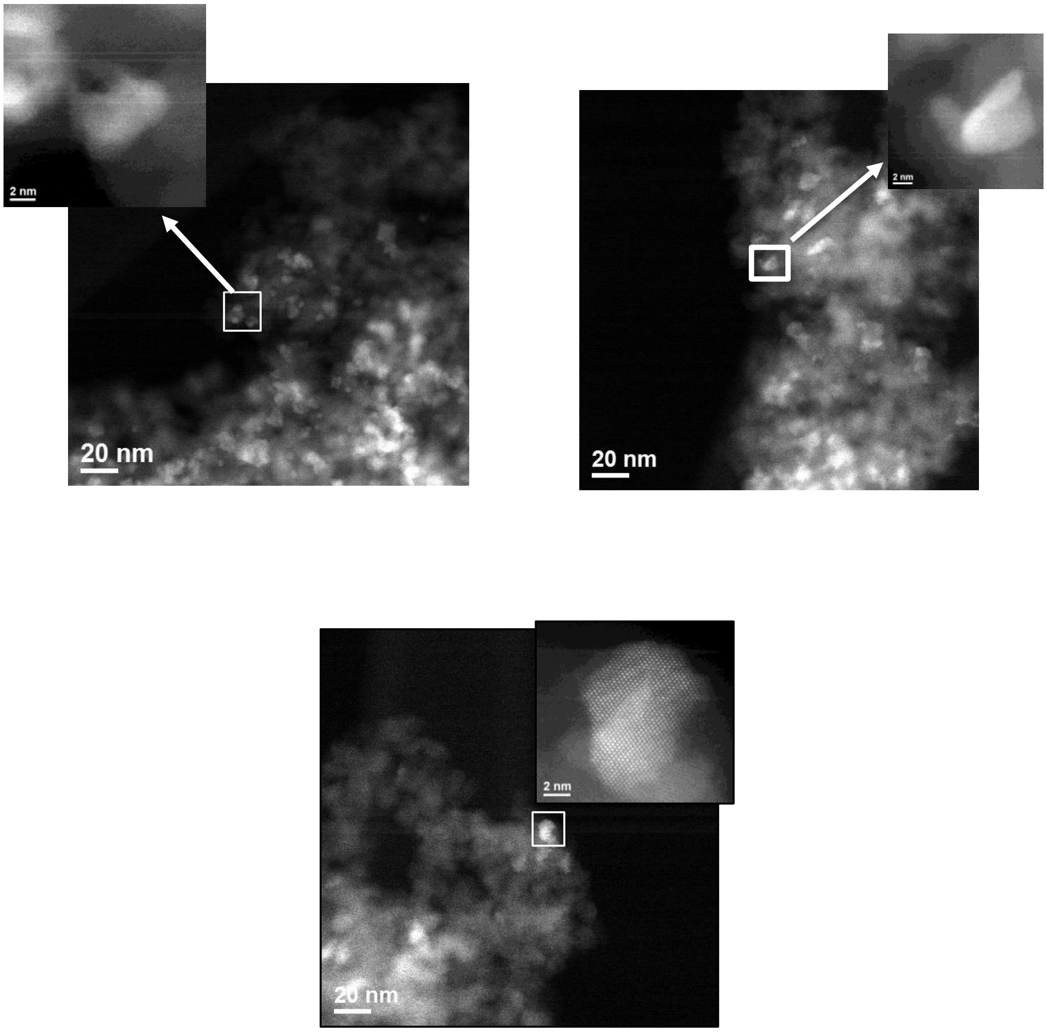

Figure S3. HAADF-STEM images of Pd/θ-alumina post NO oxidation experiment show primarily particles

Table S1. Bonding Parameters and Magnetization values of configurations in Figure S3†

Configuration	*Total Energy (eV)*	*Pd-O bonds (Å)*				*Magnetic Moment*		
		O1	*O2*	*O3*	*O4*	μ_{Total}	μ_{Mtotal}	μ_{Ototal} O1 O2 O3 O4
V	-1320.524	2.07	2.24	2.11	1.96	0.231	0.0	0.0, 0.0, 0.0, 0.0
VI	-1320.657	2.07	2.26	2.17	1.98	0.64	0.12	0.02, 0.01, 0.01, 0.13
VII	-1300.425	-	2.19	2.15	-	1.0	0.63	-, 0.14, 0.16, -
VIII	-1311.131	2.39	2.37	2.00	-	1.0	0.13	0.0, 0.00, 0.00, -
IX	-1324.047	2.42	2.48	2.07	-	-0.02	0.17	0.05, 0.00, 0.04, -

† Pd is bonded to surface oxygen O1-O4. Magnetization spread over other surface atoms is not listed in the table.

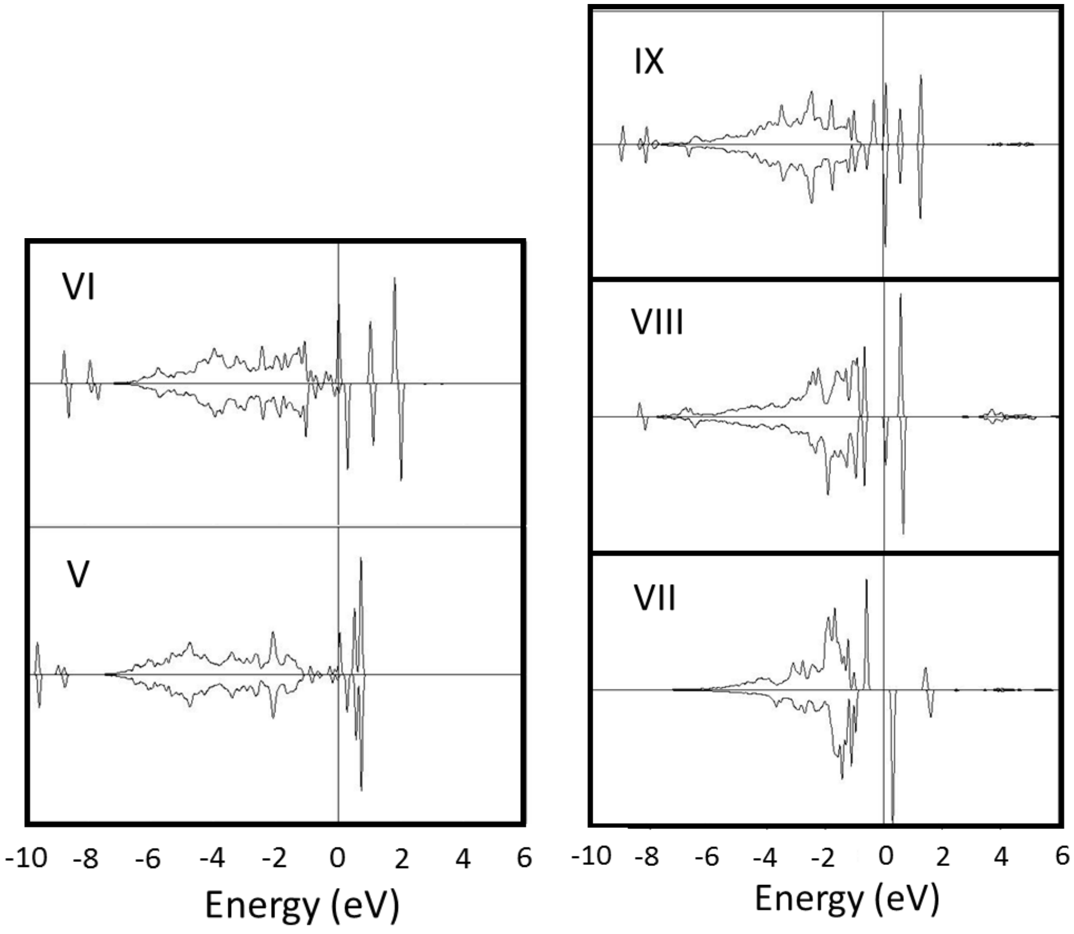

Figure S4. PDOS of d-orbitals of configurations V-IX

Table S2. Bonding Parameters and Magnetization Values of Configurations in Figure 8†

Configuration	*Total Energy (eV)*	*Pd-O bonds (Å)*		*Magnetic Moment*			
		O1	*O2*	μ_{Total}	μ_{Mtotal}	μ_{Ototal} O1	O2
III	-1326.182	2.27	2.24	0.0	-	-	-
VII	-1320.510	2.05	-	2.0	0.78	0.04	
VIII	-1329.318	2.10	-	0.0	-	-	-
IX	-1339.662	2.48	-	1.0	0.01	0.0	-
X	-1340.150	2.26	2.23	1.0	0.56	0.02	0.3
XI	-1333.948	2.36	-	1.0	0.29	0.01	

† Pd is bonded to surface oxygen O1 and O2. Magnetization spread over other surface atoms is not listed in the table.

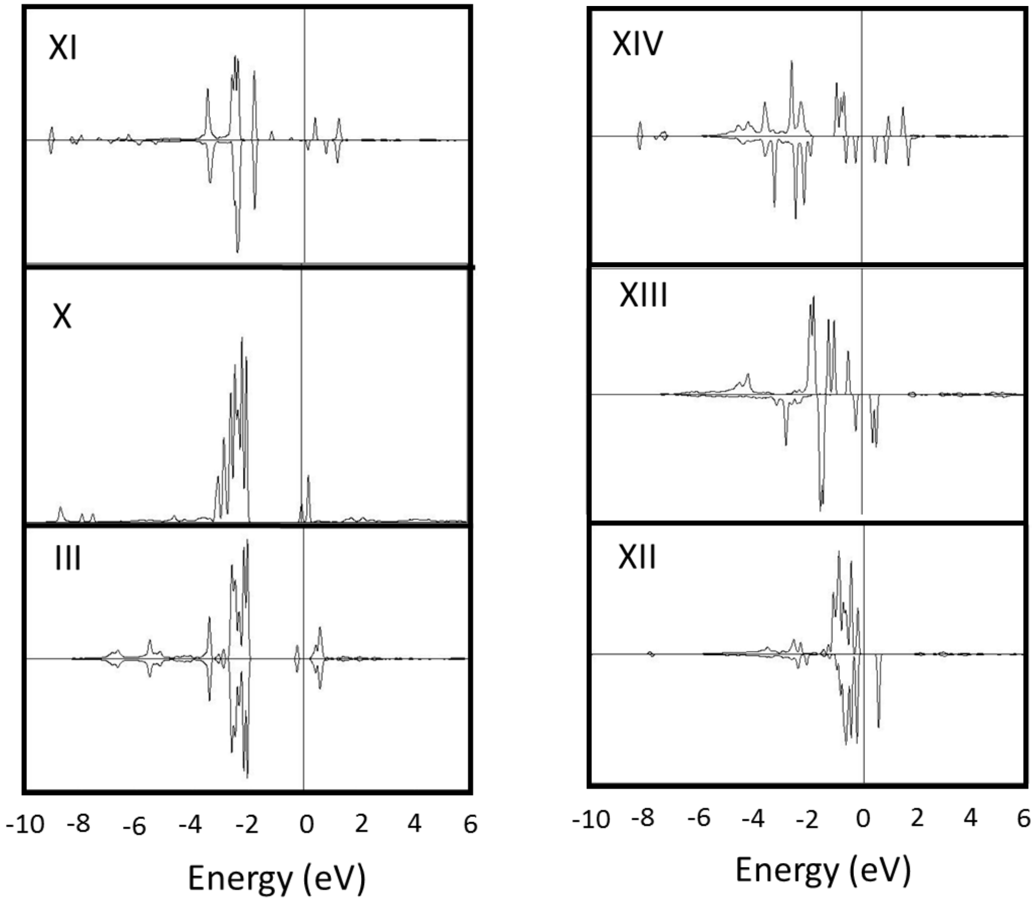

Figure S5. PDOS of d-orbitals of configurations III, X-XIV

NO Oxidation on Pd Adatom.

As discussed in the manuscript, there is no driving force for the release of NO_2 from transition state ii or from configuration XII. If NO_2 is indeed released from configuration XII, the reaction would be an endothermic process resulting in the formation of configuration XIII which can reaction with a second molecule of NO to form configuration XIV via an exothermic process (-1.45 eV). The NO bonds to Pd in configuration XIV in a bent mode with a Pd-N-O angle of 134.1° suggesting a NO^- species. The magnetization is distributed over Pd and dissociated oxygen suggesting a d^9 Pd species. Pd is bonded to one surface oxygen with Pd-O bond distance of 2.36Å. The PDOS show partially occupied dxy, dxz, and dx^2 orbitals [Figure S 5]. The loss of NO_2 from configuration XIV can lead to leads to configuration I but the energetics (exothermic by -0.01 eV) suggest that there is no driving force for this reaction also. The overall NO oxidation cycle on a single supported Pd adatom is shown in Figure S5. Total energy, Pd bond distances to surface oxygen, and magnetizations are summarized in Table S2.

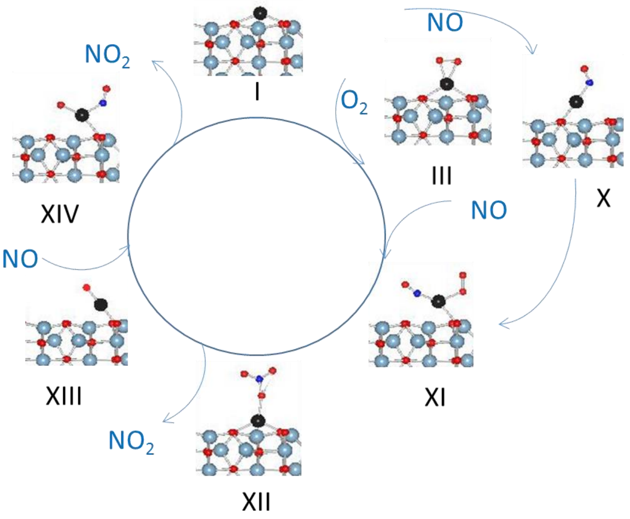

Figure S6. NO oxidation cycle on single supported Pd adatom.

The energetics of reactions in Figure 8 are summarized as follows:

*Pd (I)	+ O_2	=	*PdO_2 (III)	-1.51 eV
*Pd (I)	+ NO	=	*PdNO (X)	-1.44 eV
*PdO_2 (III)	- O	=	*PdO (XIII)	5.58 eV
*PdO_2 (III)	+ NO	=	*$Pd(O_2)(NO)$ (XI)	-1.50 eV
* $Pd(O_2)(NO)$ (XI)		=	*$Pd(NO_3)$ (XII)	-0.49 eV
*$Pd(NO_3)$ (XII)	- NO_2	=	*PdO (XIII)	1.59 eV
*PdO (XIII)	+ NO	=	*Pd(O)(NO) (XIV)	-1.46eV
*Pd(O)(NO) (XIV)	- NO_2	=	*Pd (I)	-0.01 eV

The asterisk (*) represents θ-alumina surface in the equations (and in the energetics equation in all subsections). This mechanistic pathway suggests that NO oxidation is energetically favorable on a Pd adatom on alumina.
